# CO_2_/N_2_ Gas Separation Using Pebax/ZIF-7—PSf Composite Membranes

**DOI:** 10.3390/membranes11090708

**Published:** 2021-09-14

**Authors:** Soong-Seok Yoon, Hyun-Kyung Lee, Se-Ryeong Hong

**Affiliations:** 1 Department of Chemical Engineering and Materials Science, Sangmyung University, 20 Hongjimun 2-gil, Jongno-gu, Seoul 03016, Korea; cab5836@gmail.com (S.-S.Y.); hklee@smu.ac.kr (H.-K.L.); 2 Kyedang College of General Educations, Sangmyung University, 20 Hongjimun 2-gil, Jongno-gu, Seoul 03016, Korea

**Keywords:** Pebax-2533, ZIF-7, composite membrane, CO_2_ separation

## Abstract

In this study, we mixed the zeolitic imidazolate framework-7 (ZIF-7) with poly(ether-b-amide)^®^ 2533 (Pebax-2533) and used it as a selective layer for a composite membrane. We prepared the composite membrane’s substrate using polysulfone (PSf), adjusted its pore size using polyethylene glycol (PEG), and applied polydimethylsiloxane (PDMS) to the gutter layer and the coating layer. Then, we investigated the membrane’s properties of gases by penetrating a single gas (N_2_, CO_2_) into the membrane. We identified the peaks and geometry of ZIF-7 to determine if it had been successfully synthesized. We confirmed that ZIF-7 had a BET surface area of 303 m^2^/g, a significantly high Langmuir surface area of 511 m^2^/g, and a high CO_2_/N_2_ adsorption selectivity of approximately 50. Considering the gas permeation, with ZIF-7 mixed into Pebax-2533, N_2_ permeation decreased from 2.68 GPU in a pure membrane to 0.43 GPU in the membrane with ZIF-7 25 wt%. CO_2_ permeation increased from 18.43 GPU in the pure membrane to 26.22 GPU in the ZIF-7 35 wt%. The CO_2_/N_2_ ideal selectivity increased from 6.88 in the pure membrane to 50.43 in the ZIF-7 25 wt%. Among the membranes, Pebax-2533/ZIF-7 25 wt% showed the highest permeation properties and the characteristics of CO_2_-friendly ZIF-7.

## 1. Introduction

Global warming is a persisting issue, and the Intergovernmental Panel on Climate Change (IPCC) have warned that the average temperature on earth could increase by 1.5 °C between 2030 and 2052 if the current trend continues [1]. Separation membranes are eco-friendly because no harmful substances are generated during the separation process, and they have the advantage of cost-effectiveness and high energy efficiency [2,3]. In particular, the polymer membrane has been widely researched because of its low cost and is easy to manufacture, but the associated problem is a “trade-off” between permeability and selectivity that are inversely proportional to each other [4,5,6]. The mixed-matrix membrane (MMM), with inorganic materials and a thin-film composite (TFC) membrane with a thin selective layer applied on its substrate, is a solution to overcome this problem.

MMM has properties of both polymers and inorganic materials, resulting in high mechanical and thermal strengths compared with pure polymer membranes, and has the advantage of lower-cost over pure inorganic membranes in terms of manufacturing [2]. However, because the phase separation can occur between polymers and particles, there must be a good affinity between the inorganic particles and polymers being used. The cohesion of particles or the rigidification of the polymer chain can be avoided if the affinity between polymers and inorganics is good [6]. Thus, numerous attempts have been made to improve the affinity between polymer and particles, and there have been reports of improvements in existing pure membrane permeability properties by using them in an MMM. Unlike these MMMs, in TFC membranes manufactured with thin selective layers, permeability resistance decreases and permeability increases as the membrane gets thinner [5,7,8]. In general, thinning the selective layer in the membrane results in a decrease in the mechanical strength; therefore, it is manufactured by applying it on top of porous substrates. Porous substrates have good mechanical strength, low resistance so that the permeation selectivity remains unaffected, very high permeability, and low selectivity [5,8,9]. When a selective layer is applied directly onto the substrate, the solution may permeate into the pore, causing defects in the selective layer, and obtaining a selective layer with a uniform thickness may become challenging. Therefore, a gutter layer (or an intermediate layer) is applied to protect the selective layer with little impact on the permeating selectivity and helps to maintain a uniform thickness of the selective layer [8,10]. Research has been conducted for preventing the intrusion of the applied layer by reducing the pore size of the substrate. Y. Ma et al. [11] attempted to reduce the pore size and increase the porosity using polyethylene glycol (PEG) by mixing the polysulfone (PSf) substrate with PEG of various molecular weights (400, 800, 1500, 4000, 10,000, and 20,000 Da) and found that when dissolved in the macromolecular solution, the concentration increases, and because PEG is so hydrophilic that it affects the phase transition, porosity gets higher than the existing PSf and the contact angle decreases if PEG is mixed. B. Chakrabarty et al. [12] used scanning electron microscope (SEM) observations, performed water treatment, and analyzed gas permeation after mixing three types of PEG (400, 6000, and 20,000 Da) with PSf. They found that the pore size decreased and the porosity increased with the mixing and also found that the larger the PEG molecular weight, the smaller was the pore size. When considering the pore size distribution, they found that the mean pore radius was 0.11 μm at PEG 400 Da and 0.04 μm at PEG 20,000 Da based on the NMP solvent. They also confirmed that surface porosity increased from 0.20 at PEG 400 Da to 0.37 at PEG 20,000 Da. This phenomenon occurred in both commonly used NMP and DMAc [12]. Therefore, when PEG is mixed into PSf during the membrane manufacturing, the pore size decreases and porosity increases with the increase in the molecular weight, and the mixed content must be considered. On the selective layer used on the substrate, a protective layer (or coating layer) of polydimethylsiloxane (PDMS) or poly[1-(trimethylsilyl)-1-propyne] (PTMSP) can also be applied to prevent damage such as the aging of membranes according to the characteristics of macromolecules and improve the stability by smoothening the surface. However, in the case of PTMSP, because of the drastic decline in CO_2_ permeation due to aging, PDMS is preferred as a protective layer [10,13,14].

Metal–organic frameworks (MOFs), among filling materials added into the polymer, are porous crystals produced by the bonding of the metal cluster with organic ligands, which have a wide surface area and pore volume and can be variously formed following the bonding method. Owing to the high porosity and surface area of MOFs, several studies have been conducted on catalysts, drug delivery, and adsorption [3,15,16]. MOF has coordination bonds and van der Waals bonds and characteristically has structural flexibility because of weak bonding force [5,16]. Among MOFs, zeolitic imidazolate frameworks (ZIFs) are porous substances produced by the coordinate covalent bonding of zinc or cobalt ions with organic ligands [17,18,19]. The metal–imidazolate–metal (M–IM–M) bonding angle is very similar to the Si–O–Si angle (145°) of zeolite [3,18,19], and the frame of the particles is flexible, resulting in gate-opening, which varies in pore size and structure depending on the gas, temperature, and pressure conditions [18,19,20].

The zeolitic imidazolate framework-7 (ZIF-7) is a porous assembly of zinc ions and benzimidazole that takes the form of sodalite (SOD). The pore size is approximately 3.0 Å. In the framework of particles, the benzimidazole ligand has a CO_2_-friendly property, resulting in a gate-opening phenomenon in which the angle formed by the vertical plane separating the interior of the ZIF-7 and the ligand gets larger than the conventional 48° by approximately 13°–14°, and the pore size increases up to 5.2 Å. This allows ZIF-7 to exhibit a pore size larger than the CO_2_ molecule, which increases CO_2_ permeability [3,21,22] and the ideal selectivity of CO_2_/N_2_ and CO_2_/CH_4_. N. Azizi et al. [23] prepared MMMs using a Pebax-1074 polymer mixed with ZIF-7 by content (0–25 wt%), performed CO_2_/CH_4_ gas permeation, and estimated the permeability of CO_2_ and CH_4_ to be approximately 65.82 and 8.36 Barrer in a pure membrane and 101.34 and 122.24 Barrer (the largest) in ZIF-7 10 wt%, respectively. They also confirmed that the CO_2_/CH_4_ ideal selectivity steadily increased from approximately 20.34 in a pure membrane to approximately 28.16 in ZIF-7 25 wt%. Li et al. [24] examined the single-gas permeability of CO_2_, CH_4_, and N_2_ after mixing ZIF-7 into the Pebax-1657 and compared the pure membrane with the membrane containing ZIF-7 at a high content (34 wt%) to find that CO_2_ permeability decreased from 72 to 41 Barrer, but CO_2_/N_2_ ideal selectivity increased from 34 to 105, which exceeded the Robeson upper bound reported in 2008. When Chakrabarty et al. [25] mixed the PAN-r-PEGMA67 polymer with ZIF-7 of up to 33.4 wt%, CO_2_ permeability decreased from 43 to 13 Barrer, but CO_2_/CH_4_ ideal selectivity increased from 19 to 39. Recently, J. Gao et al. [26] conducted CO_2_/N_2_ gas separation after preparing a MMM using Pebax-2533, ZIF-7, and ZIF-7 modified by attaching various kinds of a functional group; they confirmed that ZIF-7-OH exhibits good permeation properties at low content.

In this study, we used Pebax-2533 with polyamide 20 wt%, and polyether 80 wt% out of poly(ether-block-amides) (Pebax^®^)m that have good CO_2_ selective permeability as the basic material for the polymer membrane. Pebax^®^ is a copolymer thermoplastic macromolecule composed of polyether blocks bonded with polyamide blocks [23,27,28]. Polyether blocks have chain fluidity and large free-volume, whereas polyamide blocks have excellent mechanical strength [23,28,29]. In particular, polyether blocks have a strong affinity for polar molecules such as CO_2_, which promotes permeability. Therefore, this polymer is suitable for CO_2_ separation owing to the polyether group, and numerous related studies have been conducted [30,31]. We synthesized the zeolitic imidazolate framework-7 (ZIF-7), which has a high affinity for CO_2_, as a filler applied to within Pebax-2533 and confirmed material properties using FT-IR, XRD, TGA, FE-SEM, and gas adsorption capability via BET analysis of the synthesized ZIF-7. From an industrial perspective, to handle the CO_2_ generated using polymer membranes, the permeability needs to be increased; thus, the composite membrane format becomes crucial. We used a porous substrate of composite membrane format, which has PEG that supplements porosity and pore size, mixed in polysulfone (PSf), and applied a polydimethylsiloxane (PDMS) gutter layer to prevent the selective layer from penetrating the pores of the substrate, making the selective layer uniform, and used the protective layer to protect the selective layer from outside damage and to enhance surface stability. The majority of the composite membranes did not use the protective layer. According to Chakrabarty. T et al. [25], the membrane’s surface become rougher when ZIF-7 was added into the polymer. S.H. Woo et al. [32] confirmed that when comparing a membrane with a smooth surface and a membrane with a rough surface, the smooth membrane showed excellent permeation performance compared to the rough membrane. In addition, it can be relatively easy for the rough membrane to cause external physical damage. Therefore, we determined to use a protective layer. We intended to synthesize a Pebax-2533/ZIF-7 composite membrane based on the substrate made for porosity and fault prevention of the selective layer. In this composite membrane, changes in gas permeability and CO_2_/N_2_ separation are studied while varying the ZIF-7 content added to Pebax-2533. Additionally, by adding ZIF-7 up to 50 wt%, we wanted to check the limit of how far the performance of ZIF-7 could be reached in Pebax-2533, and to study the composite membrane that shows the optimal result in CO_2_/N_2_ separation.

## 2. Materials and Methods

### 2.1. Materials and Reagents

For ZIF-7 synthesis, we used 98% purity zinc nitrate hexahydrate (Zn(NO_3_)_2_·6H_2_O) and 98% purity benzimidazole reagent from Sigma-Aldrich (St. Louis, MO, USA). Additionally, we used 99.5% purity reagent N,N-dimethylformamide (DMF) from Samchun Chemicals (Seoul, Korea) as a solvent for ZIF-7 synthesis, 99.9% purity HPLC grade methanol from Carlo Erba (Val-de-Reuil, France) as a post-synthetic cleaning solvent, Solvay (Haandorpweg, Belgium) Udel^®^ P-3500 LCD pellet of polysulfone (PSf) as the material of the substrate for the composite membrane’s mechanical strength, 100% purity N-methyl-2-pyrrolidone (NMP) from Sigma-Aldrich (St. Louis, MO, USA) as a solvent, PEG of the molecular weight of 20,000 Da (Average molecular weight: 15,500–25,000 Da) from Junsei (Tokyo, Japan) as an additive of the substrate, poly(ether-block-amide) (Pebax) 2533 from Arkema (Colombes, France), solvents of 99.5% purity isopropanol and 99.0% purity n-butanol from Daejung Chemicals (Jeongwang-Dong, Korea), Sylgard 184A and Sylgard 184B as polydimethylsiloxane (PDMS) monomer and initiator from Dow Corning (Midland, MI, USA) as materials for the gutter layer and the protective layer of the composite membrane, respectively, and 95% purity n-hexane from Daejung Chemicals (Jeongwang-Dong, Korea) as PDMS’s solvent.

### 2.2. Synthesis of ZIF-7

We synthesized ZIF-7 by referring to the literature of Al-Maytalony et al. [33]. The process was as follows:

1.25 g of zinc nitrate hexahydrate (Zn(NO_3_)_2_·6H_2_O) was added in 100 mL of N,N-dimethylformamide (DMF) and stirred for 30 min to disperse. 1.54 g of benzimidazole was added in 100 mL of N,N-dimethylformamide (DMF) and stirred for 30 min to disperse. The two stirred solutions were mixed and stirred while heating at 313 K for 72 h to obtain a white suspension. The suspension was centrifuged (4200 rpm, 30 min), the mother liquid was separated at the top, and the bottom solution was washed thrice using methanol. When washing, the product (i.e., the bottom solution) and methanol were put in a round bottom flask and stirred for 3 h to ensure the removal of the solvent from the particles. After washing, the particles were dried in an oven at 363 K for 12 h.

### 2.3. Fabrication of Composite Membrane

18 wt% PSf solution was prepared by mixing PSf and NMP; the solvent was in the ratio of 18:82 (wt/wt), and heating and stirring was performed at 353 K for five hours. Then, the PSf solution was left at room temperature for 1 d to remove the gas in the solution and cool. PEG by 1 wt% of the weight of the PSf pellet was taken and put in the PSf solution; it was heated and stirred at 353 K for 6 h to prepare PSf and PEG mixture solution and left at room temperature for 1 d. The finished PSf and PEG mixture solution was cast to 200 μm thickness on a glass plate using a casting knife and immediately immersed into room-temperature distilled water (coagulating liquid). The distilled water was changed thrice in 2 h, then again in another 2 h, and again in 24 h to completely remove the PEG within the substrate. Then, it was dried at room temperature for 3 d to complete the PSf substrate membrane.

After stirring the PDMS monomer and n-hexane solvent at room temperature for 2 h, the initiator was added and stirred again at room temperature for 2 h to prepare the PDMS 0.5 wt% solution. This solution was poured sufficiently over the previously made PSf substrate, cast to 200-μm thickness using a casting knife, and dried at room temperature for 2 d to complete the gutter layer.

**Figure 1:**
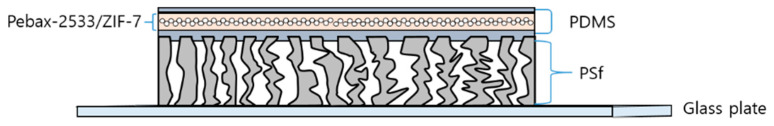
The fabrication of composite membrane.

After preparing a mixture solvent of isopropanol and n-butanol at 3:1 wt%, a 2.5 wt% Pebax-2533 solution was prepared by mixing the Pebax-2533 polymer. As much ZIF-7 as 5, 15, 25, 35, and 50 wt% of Pebax-2533 weight was separately put into the mixture solvent and then to 1-h sonication. Then, it was stirred at room temperature for 1 d for sufficient dispersion, and the prepared Pebax-2533 solution was mixed into the ZIF-7 solution. It was again put to 1-h sonication and 1-day room-temperature agitation. The Pebax-2533/ZIF-7 solution was poured over the PDMS gutter layer of the substrate, cast to 200 μm thickness using a casting knife, and dried overnight at 343 K to complete the selective layer. The protective layer for the selective layer was made using the same PDMS 0.5 wt% solution as the gutter layer, and the solution was poured on the whole surface of the membrane, tilted over the membrane for 5 s at an angle of 45°, and dried for 2 d at room temperature to prevent the damage of the selective layer. The scheme of fabricated composite membrane was indicated to Figure 1. The content of ZIF-7 applied to Pebax-2533 was calculated using the following Equation (1):

(1) $$\mathrm{Particle}\;\mathrm{loading}=\frac{Weight\;of\;Particles}{\left(Weight\;of\;Particles+Weight\;of\;Polymer\right)}\times 100\;\left(\mathrm{wt}\%\right)$$

### 2.4. Analysis Instruments

We conducted FT-IR analysis in the range from 400–4000 cm^−^^1^ using Brucker’s (Billerica, MA, USA) Vertex 70. As a powder X-ray diffraction (XRD) analyzer we used SmartLab from the Rigaku Corporation (Tokyo, Japan) under CuK-alpha, 40 kW, 2θ = 3° to 80°, 3°/min speed conditions. In BET analysis, we used Belsorp-max from the MicrotracBEL Company (Osaka, Japan) for adsorption–desorption of N_2_ and CO_2_ at room temperature and for specific surface analysis under 77 K conditions. We used Q50 from TA Instruments (Newcastle, DE, USA) for thermogravimetric analysis (TGA) to measure weight loss in the N_2_ environment in the range from 303–1073 K at the rate of 10 K/min. In the case of a differential scanning calorimeter (DSC), we used TA Q2000 from TA Instruments (Newcastle, DE, USA) to analyze the form of the film under the N_2_ environment. To check the structure of the substrate, we observed it under an accelerated voltage of 30 kV using the SEM JSM-5600LV from JEOL (Tokyo, Japan). We used a field emission SEM (FE-SEM) SU-8010 from the Hitachi Corporation (Tokyo, Japan) set at an accelerated voltage of 10 kV for analyzing ZIF-7, the surface, and the cross-section of the composite membrane. We used GPA-2001 from the SepraTek Company (Incheon, Korea) for the gas permeation measurement.

### 2.5. Gas Permeation Test

We conducted the gas permeation experiment of the Pebax-2533/ZIF-7 composite membrane under 298 K and 304 kPa conditions using 99.999% purity N_2_ and 99% purity CO_2_. The measurement was performed using a continuous flow method [34], and the gas permeating device used comprised a pressure transmitter (PT), mass flow meter (MFM), buffer tank, and membrane cell. The MFM, which measures the change in permeation speed of gas, had a capacity of 1000 SCCM. The effective area of the membrane, measured in conjunction with the membrane cell, was 12.56 cm², and the thickness of the composite membranes was found to be approximately 130 μm.

The solution-diffusion theory, Fick’s law, and Henry’s law were applied to obtain the following Equation (2) of permeability (P) for each gas:

(2) $$P_{i}=\frac{l}{A\Delta p}\frac{dV_{i}}{dt}$$

where *l* is the membrane thickness (cm), *A* is the effective area of the membrane (cm^2^), Δ*p* is the pressure difference between the top and bottom of the membrane (cmHg), *i* is the permeating gas, *V_i_* is the volume of the gas that permeated the membrane (cm^3^, STP), and *t* is the gas permeation time (s).

The diffusion coefficient of a gas passing through the membrane is expressed in Equations (3) and (4):

(3) $$D_{1/2}=\frac{l^{2}}{7.2t_{1/2}}$$

(4) $$D_{slope}=\frac{l^{2}}{5.91t_{slope}}$$

where *D*_1/2_ and *D_slope_* are diffusion coefficients for response times *t*_1/2_ and *t_slope_*, respectively, and we defined *D_slope_* as diffusivity *D*.

Permeability (P) can be calculated using diffusivity (D) and solubility (S) and is represented by Equation (5):

(5) P=D×S

The ideal selectivity (*α*) for two gases can be obtained from the following Equation (6):

(6) $$\alpha_{a/b}=\frac{P_{a}}{P_{b}}=\left(\frac{D_{a}}{D_{b}}\right)\left(\frac{S_{a}}{S_{b}}\right)$$

Here, the ratio of the permeability of gas *a* relative to gas *b* is expressed as *α_a/b_*.

## 3. Results and Discussion

### 3.1. Properties of ZIF-7 Particles

**Figure 2:**
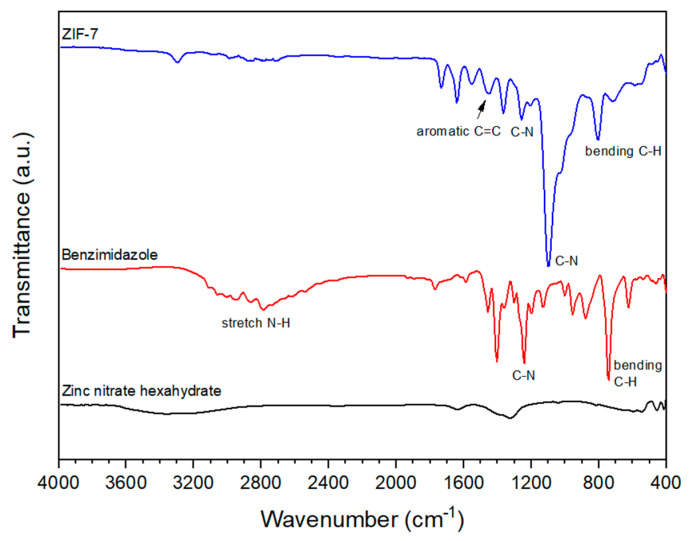
FT−IR spectra of zinc nitrate hexahydrate, benzimidazole, and ZIF-7.

Figure 2 presents the FT-IR of the raw material required for ZIF-7 synthesis and synthesized ZIF-7. We confirmed that each peak displayed in Figure 2 coincided with that in other papers [35,36,37]. A wide N-H stretch bond was identified in the IR of the raw material benzimidazole between 2500 and 3250 cm^−1^. Deprotonation of the N-H bond developed when synthesized as ZIF-7, resulting in a coordinate bond to change from the N-H bond to an N-Zn bond. This can be seen from the fact that the peak of ZIF-7 does not appear in the region indicated by the N-H bond of benzimidazole.

**Figure 3:**
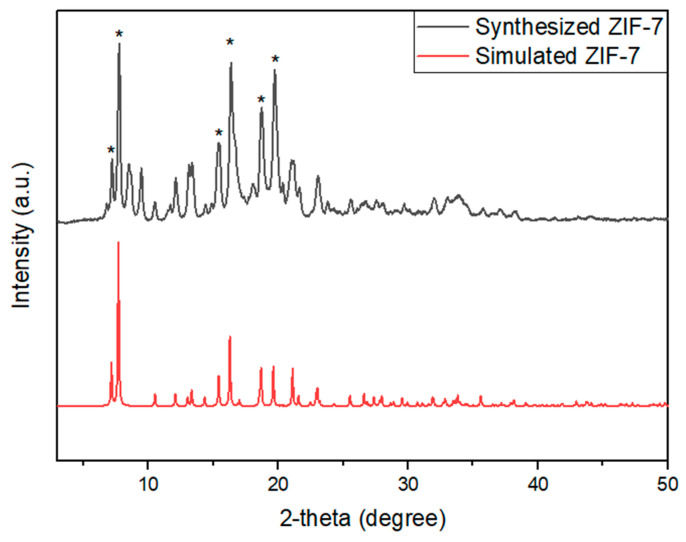
XRD patterns of synthesized and simulated ZIF-7. Asterisk (*) denotes main peaks.

In Figure 3, we compared the XRD patterns of the synthesized ZIF-7 and the ZIF-7 predicted by Park et al. [38]. Before the analysis, we dried them at 353 K for 6 h to remove the residual moisture inside. From the XRD pattern, we confirmed that the intensity of the XRD pattern of the ZIF-7 synthesized in this study at the main peaks of 7.2°, 7.7°, 15.4°, 16.4°, 18.7°, and 19.7° is almost identical to that of the XRD pattern in the literature. Presented main peaks were illustrated in the Figure 3 as an asterisk.

**Figure 4:**
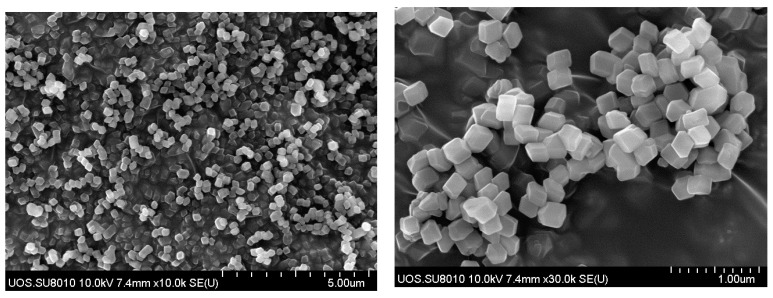
FE-SEM images of synthesized ZIF-7.

Figure 4 are FE-SEM images of the synthesized ZIF-7. The synthesized ZIF-7 was very uniform in terms of its particle size, as reported in the literature. The size was found to be approximately 130–200 nm. The particle sizes are believed to vary depending on the ratio of zinc nitrate hexahydrate and benzimidazole after their synthesis [22,39].

**Figure 5:**
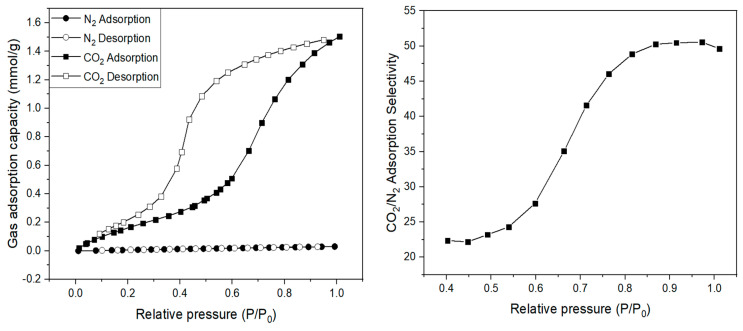
N_2_ and CO_2_ adsorption/desorption isotherms of the synthesized ZIF-7 at 293 K.

Figure 5 is the result of the BET analysis of the synthesized ZIF-7. Prior to analysis, we removed the solvent remaining in the particles by heating and vacuum treating at 473 K for 12 h. The adsorption amount of each gas at the relative pressure from the 0–1 atm (101 kPa) range indicates that N_2_ gas was almost unadsorbed but CO_2_ was adsorbed in a significant amount. The gate-opening of the ZIF-7 particles occurred near the relative pressure of 0.59 (approximately 60 kPa), which resulted in a rapid increase in the CO_2_ adsorption amount. X. Wu et al. [39] also reported that gate-opening occurred at the same pressure, resulting in considerable CO_2_ adsorption. This indicates that the ZIF-7 particles are particularly selective in gate-opening with CO_2_ gas as compared to N_2_. The CO_2_/N_2_ adsorption selectivity calculated using the adsorption amount showed an increase with increasing pressure, and at 101 kPa corresponding to 1 atm, it showed a significantly high adsorption selectivity of approximately 50. Thus, we believe that ZIF-7 particles are effective for CO_2_/N_2_ separation from CO_2_ and N_2_ adsorption amount. The Langmuir surface area and BET surface area measured at 77 K in N_2_ environments were measured to be 511 m^2^/g and 303 m^2^/g, similar to the values in other works of the literature [15,24,39,40,41], which were summarized in Table 1. Interestingly, the values of Langmuir surface area and BET surface area are confirmed as being very different. This is because of the different calculation methods for Langmuir surface area and BET surface area. They use the same value that measured the adsorption amount, but in the case of Langmuir surface area, it is assumed that internal adsorption is achieved only as a single layer. However, in the case of BET surface area, it is assumed that adsorption is possible by stacking with multiple layers. This is the reason why the two values are not the same, and generally, the Langmuir surface area has a greater value [42].

### 3.2. Properties of Composite Membranes

**Figure 6:**
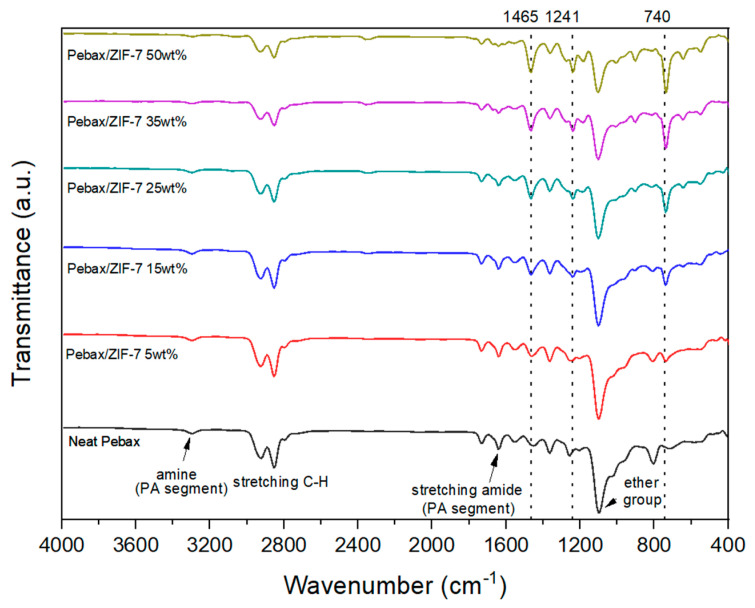
FT−IR spectra of Pebax-2533/ZIF-7 composite membranes.

Figure 6 shows an FT-IR graph of the composite membranes. The characteristic peaks seen in pure Pebax-2533 are 1103 cm^−^^1^, 1643 cm^−^^1^, 1735 cm^−^^1^, and 3300 cm^−^^1^, which correspond to (-C-O-C-), stretching vibration amide (H-N-C=O-), saturated esters (-C=O), and amine (-N-H), respectively. They were almost identical to the reports in the literature [43,44,45]. As the ZIF-7 content increased, the characteristic peaks in ZIF-7 gradually increased at 740 cm^−^^1^, 1241 cm^−^^1^, and 1465 cm^−^^1^.

**Figure 7:**
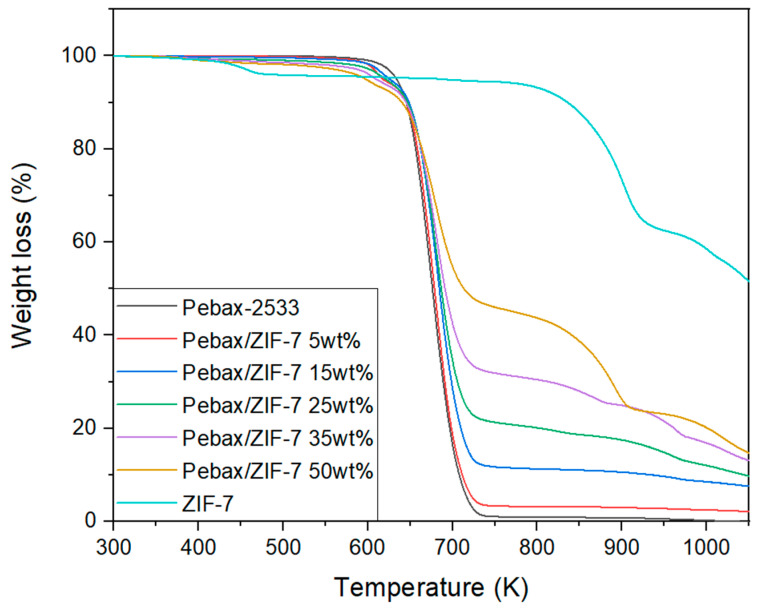
TGA thermograms of Pebax-2533/ZIF-7 composite membranes.

Figure 7 illustrates the TGA results of the ZIF-7 and Pebax-2533/ZIF-7 composite membranes. The TGA curve of ZIF-7 indicates an approximately 5% decrease in weight near 473 K for the first time, which is possibly caused by the vaporization of residual solvents and moisture contained in ZIF-7. Then, a second weight decrease begins occurring near 833 K, which is believed to be caused by the structure that collapses as the bonds of ZIF-7 break, leaving only zinc oxide (ZnO). W. Cai et al. [46] also reported a similar break, although there were some differences depending on the gas environment. From the point of view of the starting point of decomposition, the start point of weight loss was slightly faster as ZIF-7 was added, whereas the point of rapid weight loss was approximately 645 K for pure Pebax-2533 and 662 K for Pebax-2533/ZIF-7 50 wt%. Furthermore, looking at the point of weight loss when the reduction temporally ended (700–750 K), it seems that the rate of weight loss according to increasing temperature gradually decreased as the ZIF-7 was added. This indicates that mixing with ZIF-7 improves the thermal property and the number of materials remaining after combustion increases gradually with the ZIF-7 content.

Using the DSC results of the Pebax-2533/ZIF-7 membrane, we identified the changes in the melting point and degree of crystallization (Table 2 and Table 3). Pure Pebax-2533 is a copolymer of PTMO and PA-12, whose melting points were found to be approximately 288 K and 407 K, respectively; similar values were identified by Z. Dai et al. [47] and R. Casadei et al. [48]. With ZIF-7 mixed into Pebax-2533, the melting point identified at the PTMO peak increased to approximately 294 K up to ZIF-7 35 wt% and decreased sharply at 50 wt%. Furthermore, the melting enthalpy of pure Pebax-2533 was similar to the values reported by J. Kim et al. [31], and it was shown to decrease gradually with mixing with ZIF-7; we calculated the degree of crystallization using Equation (7) for a single substance and Equation (8) for the whole substance. The heat of fusion (Δ*H_m_*°) of PTMO and PA-12 was found to be 220 J/g [31,49] and 209 J/g [49,50], respectively. The degree of crystallization was calculated to be approximately 19 with pure Pebax-2533 and 8 with ZIF-7 50 wt%, which decreased with the ZIF-7 content:

(7) $$X_{c}=\frac{\Delta H_{m}\;\left(polymer\right)}{\Delta H_{m}^{{}^{\circ}}\;\left(Pure\;polymer\right)}\times 100\;\left(\%\right)$$

(8) $$X_{c\;\left(total\right)}=\frac{\Delta H_{m\;\left(PTMO\right)}+\Delta H_{m\;\left(PA-12\right)}}{\left(\Delta H_{m\;\left(pure\;PTMO\right)}^{{}^{\circ}}\times 0.8\right)+\left(\Delta H_{m\;\left(pure\;PA-12\right)}^{{}^{\circ}}\times 0.2\right)}\times 100\;\left(\%\right)$$

**Figure 8:**
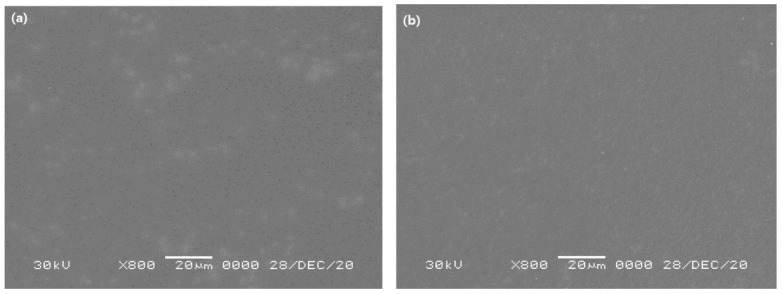
SEM images of the surface of (**a**) PSf and (**b**) PSf + PEG substrate.

**Figure 9:**
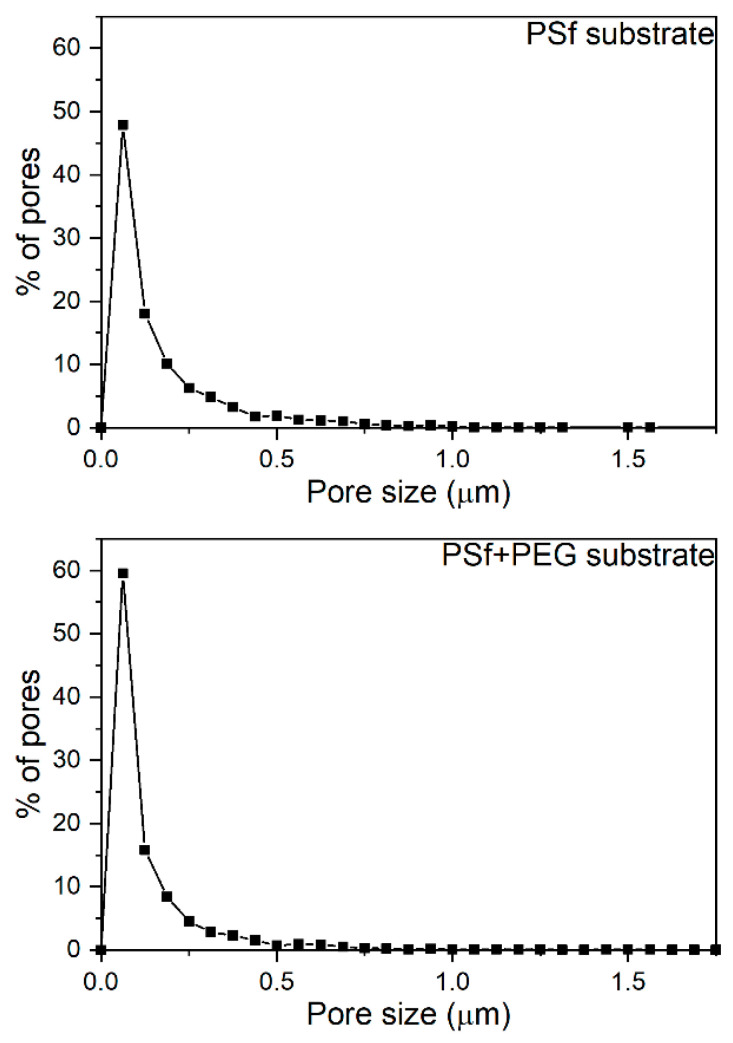
The pore size distribution of PSf and PSf + PEG substrate using ImageJ program.

**Figure 10:**
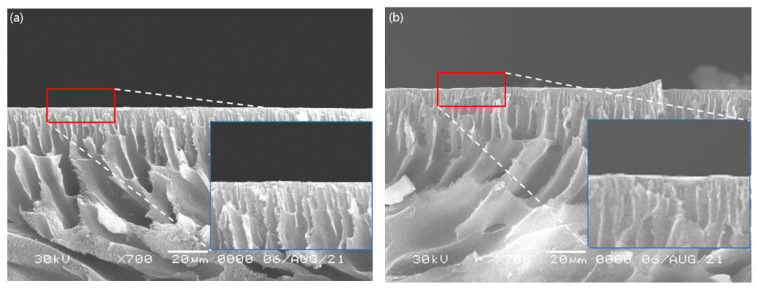
SEM images of the cross-section of (**a**) PSf and (**b**) PSf + PEG substrate.

Figure 8 shows surface SEM images of the substrates made of PSf and by adding PEG 1 wt% to PSf (PSf + PEG), in which the pore size in the PSf + PEG substrate is smaller than that in the PSf substrate. We numerically converted the surface pore sizes using the ImageJ program provided by the National Institute of Health (NIH) in the USA; the results are shown in Figure 9. According to the results obtained using ImageJ, in the case of the pure PSf substrate, the average pore size was found to be approximately 0.18 μm, with a proportion of the smallest identified pore size to be approximately 48%, whereas the average pore size of the PSf + PEG substrate was approximately 0.15 μm, which is smaller than that of the PSf substrate, with a proportion of the smallest pore size of approximately 60%. Based on this information, we could find that when the substrate is made of PSf with added PEG, porosity increases with reduced pore size, which is similar to results reported in the literature [11,12]. Figure 10 shows a finger-like structure in the SEM cross-section images of the substrate, and because the difference in pore size between the PSf and PSf + PEG substrates was too small (0.03 μm), the difference in pore size was not seen significantly in the cross-section SEM images.

**Figure 11:**
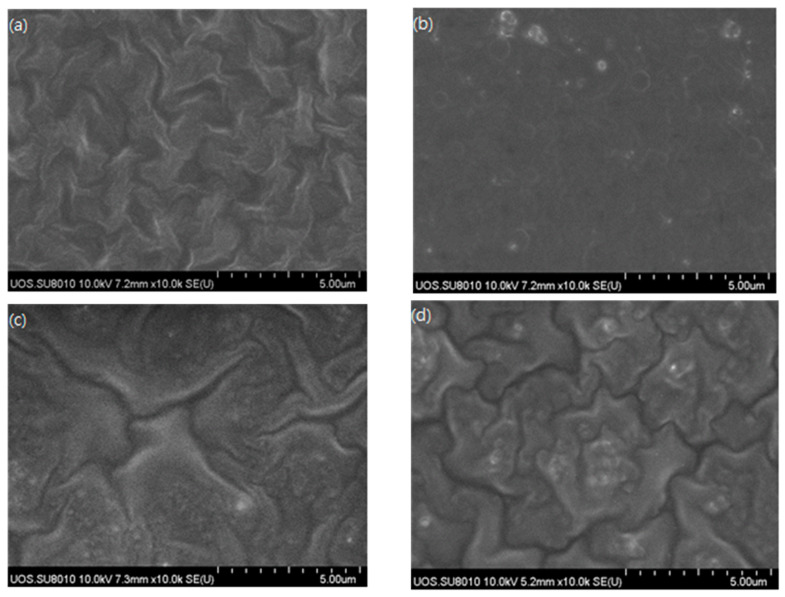
FE-SEM images of the surface of (**a**) Pebax-2533/ZIF-7 0 wt%, (**b**) 5 wt%, (**c**) 25 wt%, and (**d**) 35 wt% composite membranes.

**Figure 12:**
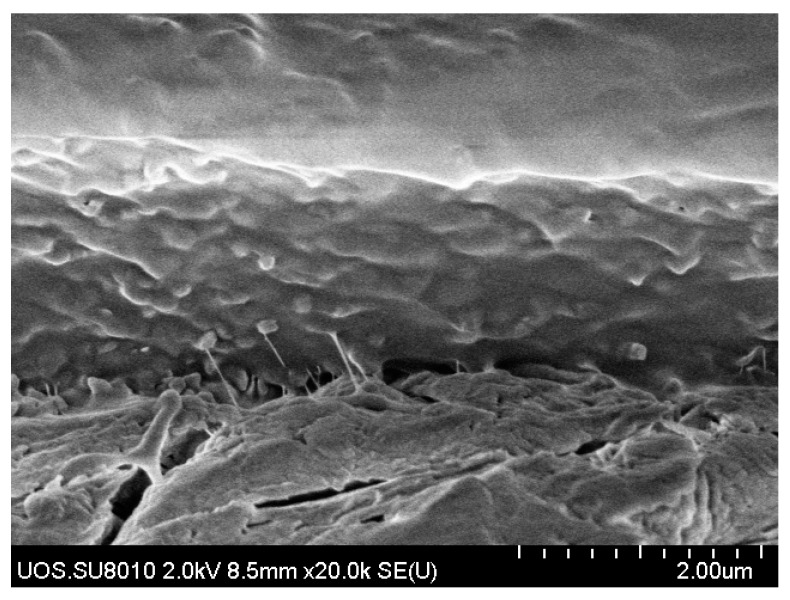
FE-SEM images of the cross-section of Pebax-2533/ZIF-7 25 wt% composite membrane.

Figure 11 shows representative FE-SEM photographs of the surface of the Pebax-2533/ZIF-7 composite membrane. The surface is not clearly visible because we formed a protective layer of PDMS in the outermost area. The pure Pebax-2533 composite membrane has a fairly neat surface. However, with the ZIF-7 mixed in, coagulated shapes are identified, and the surface is found to gradually become rough. This phenomenon was also identified by Chakrabarty et al. [25], especially in the ZIF-7 35 wt% composite membrane, in which the coagulated particles were clearly identified. The occurrence of coagulated particles means that the ideal content of filler material added in the selective layer of the composite membrane has been exceeded, which may adversely affect the interaction between the gas and the particles during gas permeation as the membrane becomes unstable and affects the dispersion of the particles. Figure 12 shows the relatively well-dispersed ZIF-7 particles in the polymer of the ZIF-7 25 wt% composite membrane.

### 3.3. Gas Permeation Properties of Composite Membranes

In this study, we prepared the Pebax-2533/ZIF-7 composite membrane and analyzed gas permeation under 298 K and 304 kPa conditions using single gases N_2_ and CO_2_. Prior to preparing the composite membrane, we found that the mix-retaining power of the Pebax-2533/ZIF-7 mix solution was so effective that the particles took more than a week to separate from the polymer in the solution. Such good mix-retaining power has also been identified by K. Xie et al. [51].

**Figure 13:**
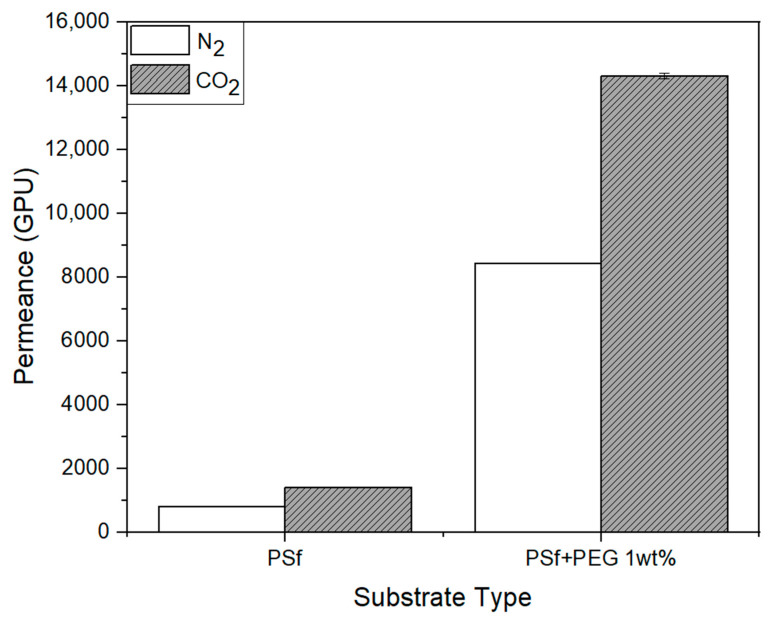
Gas permeance of PSf and PSf + PEG 1 wt% substates.

Figure 13 and Table 4 show the result of the gas permeation of the substrate used in the Pebax-2533/ZIF-7 composite membrane. As shown in Figure 8 and Figure 9, with PEG mixed within PSf, pore size decreased and porosity increased in the substrate. Before conducting this study, we had investigated how this phenomenon affects the gas permeation in advance. In the PSf + PEG substrate made by adding PEG to PSf, gas permeation of N_2_ and CO_2_ was much higher than that in the substrate made of PSf alone, and CO_2_/N_2_ ideal selectivity was also closer to 1. Y. Ma et al. [11] used PEG for preparing the substrate for a water treatment study and found an increase in permeability. Moreover, in this study, gas permeation increased significantly in the PSf + PEG substrate, possibly because of the greater porosity than that in the PSf substrate, as explained earlier using Figure 9. To summarize, we presume the following: The porosity of the structure of the PSf + PEG substrate has increased. Because the PEG used in the substrate is hydrophilic, it went through a phase transition and leaked into the water when the substrate was made. Therefore, the structure of the PSf + PEG substrate changed to be more porous than a PSf substrate. Because of this porosity, it became more gas-permeable; therefore, considering the substrate surface SEM images and pore distribution diagram of Figure 8 and Figure 9, the PSf + PEG substrate had smaller pores and increased porosity than PSf substrate. To some extent, the substrate appears to be able to compensate for defects caused by the selective layer solution being absorbed into the substrate while not affecting the gas permeating property of the selective layer in the composite membrane.

**Figure 14:**
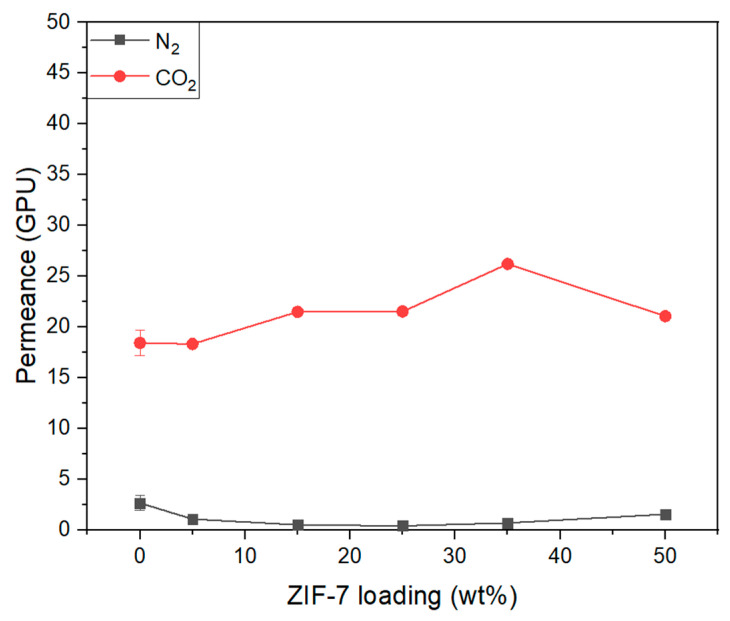
Gas permeance of Pebax-2533/ZIF-7 composite membranes.

**Figure 15:**
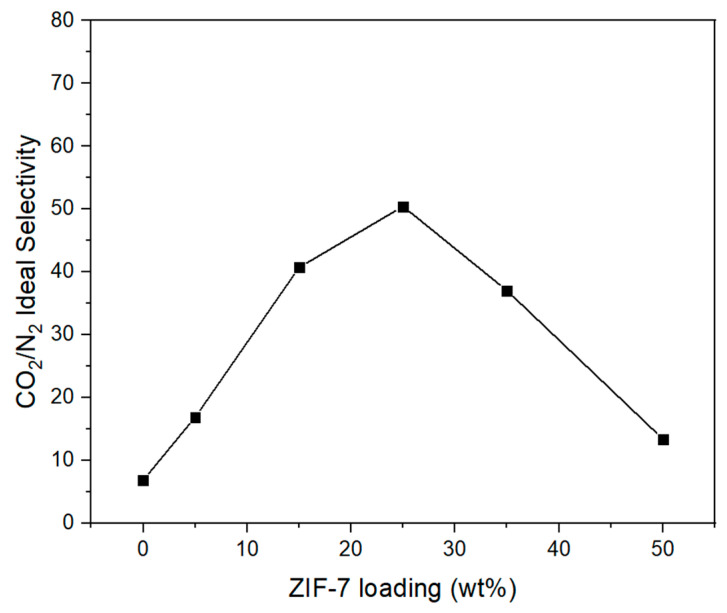
CO_2_/N_2_ ideal selectivity of Pebax-2533/ZIF-7 composite membranes.

Figure 14 shows the result of the gas permeation of the Pebax-2533/ZIF-7 composite membrane prepared based on the PSf + PEG substrate. As shown in Figure 14, N_2_ permeation of the Pebax-2533/ZIF-7 composite membranes decreased from approximately 2.68 to 0.43 GPU with up to ZIF-7 25 wt% as the ZIF-7 content increased, and slightly increased with larger content. However, CO_2_ permeation tended to increase with up to ZIF-7 35 wt% but decreased rapidly at ZIF-7 50 wt%. Figure 15 shows the CO_2_/N_2_ ideal selectivity of the Pebax-2533/ZIF-7 composite membranes. In the case of a pure Pebax-2533 membrane, it showed a tendency to increase from approximately 6.88 to 50.43 with up to the ZIF-7 25 wt% and gradually decreased with the subsequent content. The pore size of the ZIF-7 is approximately 3.0 Å, making it difficult for N_2_ with a kinetic diameter of 3.64 Å to permeate. However, gate-opening occurs for polar gases such as CO_2_, increasing the pore size of ZIF-7 up to 5.2 Å and facilitating the permeation of CO_2_ with a kinetic diameter of 3.3 Å, resulting in an increase in the permeability of CO_2_. This effect resulted in an increase in CO_2_/N_2_ ideal selectivity, as shown in Figure 15, and the largest CO_2_/N_2_ ideal selectivity at ZIF-7 25 wt%. However, starting at ZIF-7 35 wt%, CO_2_/N_2_ ideal selectivity significantly decreased with lower CO_2_ permeation, which was presumed to be the result of an excess of ZIF-7 particles being mixed, causing ZIF-7 particles to coagulate and the size of the cavity between Pebax-2533 and ZIF-7 aggregation to increase. With the addition of ZIF-7, ZIF’s strong adsorption of CO_2_ is presumed to have negatively affected the CO_2_/N_2_ gas separation. This complex phenomenon possibly resulted in significantly lower CO_2_ permeability characteristics with ZIF-7 35 wt% and above. Detailed values of Figure 14 and Figure 15 were illustrated as Table 5. Table 5 represented the thickness of selective layer, permeability and selectivity of Pebax-2533/ZIF-7 composite membranes.

Because the gas permeability is expressed as P=D×S, as shown in Equation (5), we intended to understand the impact of the diffusivity (D) and solubility (S) on the gas permeability of each composite membrane prepared in this study. In general, the gas diffusivity tends to increase as the size of the penetrant decreases, the free-volume of the polymer increases, the fluidity of the polymer chain increases, and the interaction between the penetrant gas and the polymer decreases. In contrast, the higher the condensability of the penetrant gas and the greater the interaction between the penetrant gas and the polymer, the more likely is the gas solubility to increase [27].

ZIF-7 used in this study has a gate-opening phenomenon in which phase transition occurs for CO_2_, light hydrocarbons (ethane, ethylene, and propane), etc. [3,18,22]. This is a change in the angle of the skeleton formed by the interaction of the ligands (benzimidazole ligand, Bim) of ZIF-7 has for the gases above. The pore size increases from the existing 3.0 Å to a maximum of 5.2 Å [3,39]. The CO_2_ kinetic diameter (3.3 Å) is sufficiently small to pass through the pore increased by the gate-opening effect, but N_2_, having the kinetic diameter of 3.64 Å but no gate-opening effect, makes passing difficult for ZIF-7 particles. P. Zhao et al. [21] suggested that this gate-opening phenomenon may lead to an increase in CO_2_ affinity due to the change in the electrostatic field brought about inside the particles. Several other studies [52,53] have shown that ZIF-7 has such high affinity while interacting with CO_2_ that it causes the CO_2_ solubility to increase. B.A. Al-Maythalony et al. [33] studied the gas permeability characteristics after mixing poly(ether imide) (PEI) with ZIF-7 and modified ZIF-7, and presented the tendency of gas solubility increasing and diffusivity decreasing in the MMM compared with the pure membrane. In particular, in the case of CO_2_ with mixed ZIF-7, diffusivity decreased to 1/8, but solubility increased 23 times.

**Figure 16:**
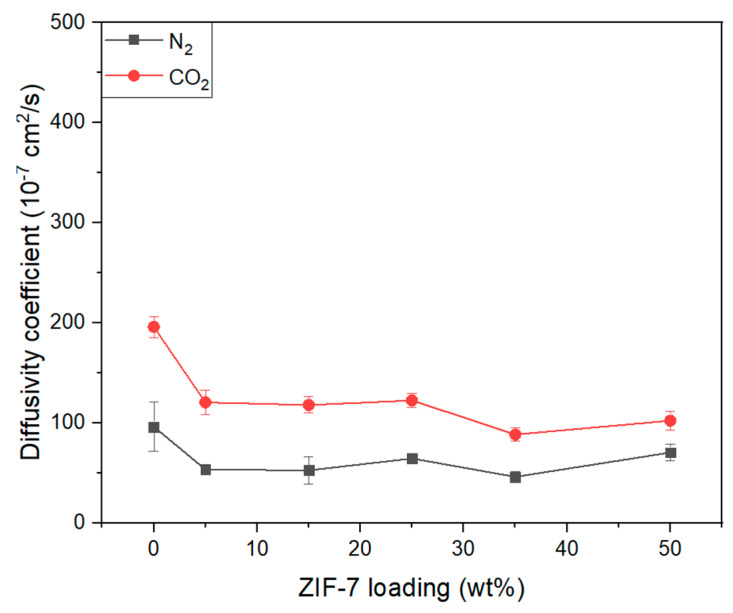
CO_2_ and N_2_ diffusivity coefficient of Pebax-2533/ZIF-7 composite membranes.

**Figure 17:**
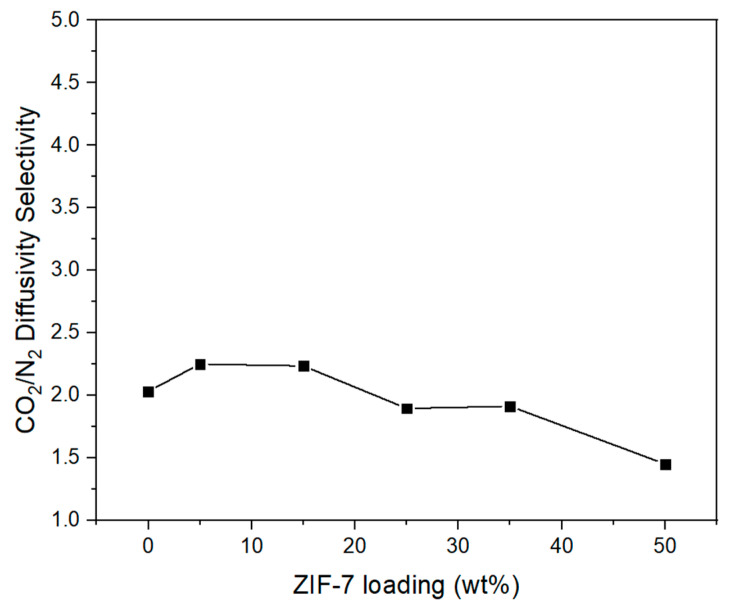
CO_2_/N_2_ diffusivity selectivity of Pebax-2533/ZIF-7 composite membranes.

Figure 16 illustrates the diffusivity of CO_2_ and N_2_ gases in the Pebax-2533/ZIF-7 composite membranes, in which the diffusivity of CO_2_ in the pure composite membrane was greater than that of N_2_. This is presumed to be due to the fact noted earlier, i.e., the kinetic diameter of CO_2_ is smaller than that of N_2_ and the pore grows bigger from the gate-opening phenomenon of ZIF-7 toward CO_2_. The overall tendency was that the diffusivity of both N_2_ and CO_2_ decreased with ZIF-7 mixed up to ZIF-7 35 wt%. A similar tendency was reported by M. Pazirofteh et al. [54] and B.A. Al-Maythalony et al. [33]. The reason for the decrease in diffusivity with the mixing of ZIF-7 is that the probability of diffusion problems increases as particles are mixed within the polymer membrane. Diffusion into particles can be described in three different ways, ideally spreading in and out of the particles, reflecting from the surface, and flowing down [55]. The diffusivity further decreases if there is not an ideal diffusion because the diffusion pathway becomes very complicated, and the diffusion pathway or average diffusion length significantly increases with the increase in the particle content in the MMM. However, with ZIF-7 35 wt%, there was a slight increase in diffusivity, possibly because the aggregation of ZIF-7 increased the size of the cavity between the interfaces of particles and the polymer, which facilitated the gas permeation to this space. Figure 17 illustrates CO_2_/N_2_ diffusion selectivity for composite membranes, where its slight increase at the initial ZIF-7 5 wt% is presumed to be because of the gate-opening phenomenon of ZIF-7 having a more significant effect on CO_2_ separation. However, considering the overall tendency with the contents thereafter, it seems that ZIF-7 did not significantly affect the selective diffusion because of the decreased diffusion selectivity. The reason for the decrease in diffusion selectivity is that the diffusivity decrease of CO_2_ works more significantly than that of N_2_, which is possibly because of ZIF-7’s strong adsorption of CO_2_ with its affinity for CO_2_, resulting in a further decrease in diffusivity. Furthermore, the kinetic diameter of N_2_ is larger than CO_2_ and originally has a lower diffusivity, while being not significantly affected by the increase in ZIF-7. However, CO_2_ has a relatively small kinetic diameter compared to N_2_, and because it is strongly adsorbed by ZIF-7, CO_2_ appears to have been greatly affected by the increase in the ZIF-7 content. Considering the BET adsorption and desorption analysis (Figure 5) of this study and in the literature [18], ZIF-7 showed high CO_2_ adsorption. Moreover, L. Zhang et al. [53], with the gas separation simulation of a MMM using polybenzimidazole (PBI) and ZIF-7, predicted the diffusivity of CO_2_ to decrease when ZIF-7 is added and explained that it was because most of the CO_2_ adsorbed by ZIF-7 reduced the diffusivity of CO_2_. Detailed values of Figure 16 and Figure 17 were indicated at the Table 6.

**Figure 18:**
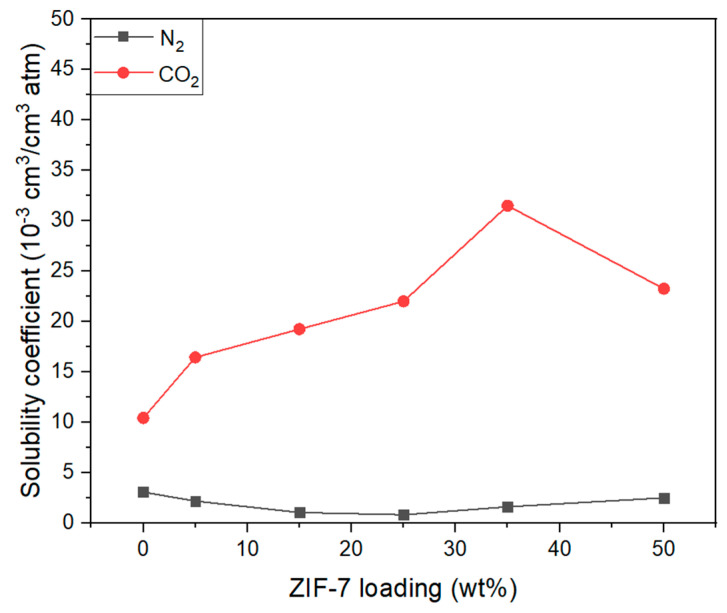
CO_2_ and N_2_ solubility coefficient of Pebax-2533/ZIF-7 composite membranes.

**Figure 19:**
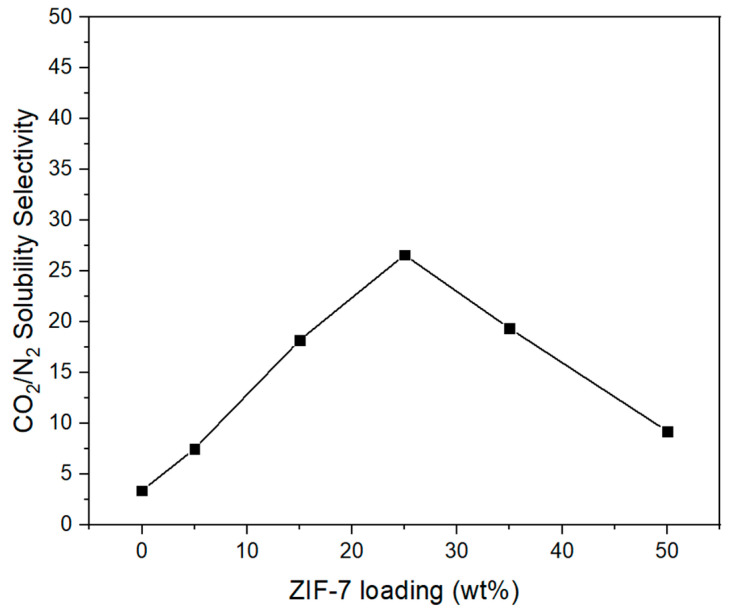
CO_2_/N_2_ solubility selectivity of Pebax-2533/ZIF-7 composite membranes.

Figure 18 shows an illustration of the solubility of CO_2_ and N_2_ gases in the Pebax-2533/ZIF-7 composite membranes, in which the CO_2_ solubility varies more significantly with increasing ZIF-7 content compared to N_2_ solubility. First, the increase in solubility up to ZIF-7 35 wt% is assumed to be due to the very significant interaction between CO_2_ and the benzimidazole ring in ZIF-7, the large space surrounded by organic linkers, and the interaction between Zn atoms in ZIF-7 and CO_2_ molecules [53]. However, with higher levels of content, the aggregation of ZIF-7 increases, and the properties of the particle reduce, resulting in a more significant aggregation effect than the interaction between the particles and gases, which is presumed to have resulted in the decrease in CO_2_ solubility. Figure 19 illustrates the CO_2_/N_2_ solubility selectivity of composite membranes where, with ZIF-7 25 wt%, the characteristics of ZIF-7 having an affinity for CO_2,_ were best demonstrated. Similar increases in CO_2_ solubility and CO_2_/N_2_ solubility selectivity were also shown by B.A. Al-Maythalony et al. [33]. Furthermore, the CO_2_/N_2_ selectivity in the composite membrane shown in Figure 15 seems to have been more significantly affected by solubility selectivity than by CO_2_/N_2_ diffusion selectivity, with ZIF-7 particles significantly affecting solubility, resulting in improved CO_2_/N_2_ ideal selectivity. Detailed values of Figure 18 and Figure 19 were indicated at the Table 7.

**Figure 20:**
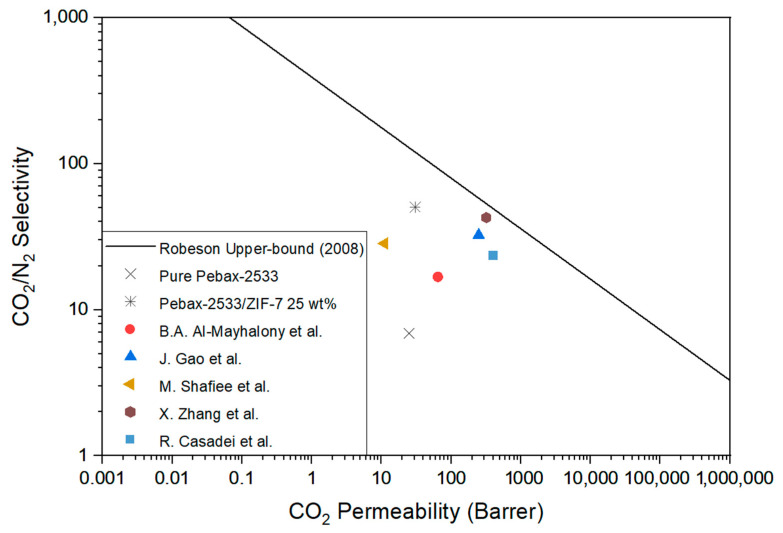
Gas permeation of pure Pebax-2533, Pebax-2533/ZIF-7 25 wt% composite membrane, and various membranes with Robeson upper bound.

Figure 20 shows the gas permeability characteristics of the Pebax-2533/ZIF-7 25 wt% composite membrane, which showed optimal performance when gas permeation and ideal selectivity were considered in this study, along with the other literature, in Robeson upper bound. The Pebax-2533/ZIF-7 25 wt% composite membrane was prepared by incorporating PEG into PSf to reduce the pore size of the support and using PDMS as a protective layer. As a result, the increase in the roughness of the selective layer due to the incorporation of a large amount of ZIF-7 and the defects caused by absorption into the pores of the support layer were reduced as much as possible. Therefore, it can be said that it is the separation membrane in which the characteristic of ZIF-7, which is friendly to CO_2_, is best expressed in the polymer. When we compared the permeation properties of the Pebax-2533/ZIF-7 25 wt% composite membrane with the optimal performance results reported in the other literature (Figure 20 and Table 8), we found improved or similar gas permeation properties.

## 4. Conclusions

We prepared composite membranes by adding 5, 15, 25, 35, and 50 wt% of ZIF-7 to Pebax-2533 and studied their gas permeability based on the content. To make the substrate to be used in the composite membrane, we mixed PEG 20000 by 1 wt% in PSf solution to reduce the surface pore size and increase porosity for improving the substrate performance. Moreover, by applying PDMS to the gutter layer and coating layer within the composite membrane, we reduced the defects in the selective layer and protected the surface of the membrane from getting rough when a large amount of ZIF-7 was added. By adding ZIF-7 up to 50 wt%, we wanted to confirm where the performance of ZIF-7 reached from Pebax-2533, where the threshold was, and study the composite membranes that show optimal permeability properties in terms of CO_2_/N_2_ separation.

Compared with the reports in the relevant literature, we found that the ZIF-7 prepared using FT-IR, XRD, FE-SEM, BET, and TGA to be well-synthesized, confirming high CO_2_/N_2_ adsorption selectivity and thermal stability. In gas permeation experiments conducted at 298 K and 304 kPa, the CO_2_ permeability of the Pebax-2533/ZIF-7 composite membrane, unlike the N_2_ gas, increased with up to ZIF-7 35 wt% and decreased rapidly with the subsequent contents. The CO_2_/N_2_ ideal selectivity gradually increased as the ZIF-7 content increased, showing a value of 50.43 with ZIF-7 25 wt%, and with the subsequent contents, a decreasing trend was observed. With ZIF-7 25 wt%, the maximum CO_2_/N_2_ ideal selectivity appeared, and the CO_2_ affinity of ZIF-7 was best shown, and with subsequent contents, the performance of ZIF-7 gradually deteriorated with aggregation. By checking the changes in diffusivity and solubility that affect gas permeation according to each content, it was confirmed that CO_2_/N_2_ ideal selectivity was predominately affected by solubility. We believe that this work provides some indications about the effects of ZIFs on CO_2_/N_2_ separation by using Pebax-2533-based membranes.

## Figures and Tables

**Table 1:** BET and Langmuir surface area of ZIF-7 with references.

Condition	BET Surface Area (m^2^/g)	Langmuir Surface Area (m^2^/g)	Reference
N_2_, 77 K	303	511	This work
	312	335	[39]
	282	527	[15]
	362	484	[24]
	380	-	[40]
	405 (Simulated)	-	[41]

**Table 2:** Melting temperature and melting enthalpy of Pebax-2533/ZIF-7 composite membranes.

Membranes	T_m (*PTMO*)_ [K]	Melting Enthalpy _(*PTMO*)_ [J/g]	T_m (_*_PA_*_− 12)_ [K]	Melting Enthalpy _(*PA* − 12)_ [J/g]	Reference
Pebax-2533	288	-	413	-	[47]
	283	-	403	-	[48]
	-	31	-	5	[31]
	288	32	407	6	This work
Pebax-2533/ZIF-7 5 wt%	289	31	405	5	
Pebax-2533/ZIF-7 15 wt%	290	26	406	4	
Pebax-2533/ZIF-7 25 wt%	291	24	405	6	
Pebax-2533/ZIF-7 35 wt%	294	22	408	3	
Pebax-2533/ZIF-7 50 wt%	286	13	409	2	

**Table 3:** Crystallinity of Pebax-2533/ZIF-7 composite membranes.

Membranes	*X_c_*_(*PTMO*)_ [%]	*X_c_*_(*PA* − 12)_ [%]	*X_c_* [%]
Pebax-2533	16	3	19
Pebax-2533/ZIF-7 5 wt%	15	2	18
Pebax-2533/ZIF-7 15 wt%	13	2	15
Pebax-2533/ZIF-7 25 wt%	12	3	15
Pebax-2533/ZIF-7 35 wt%	11	2	13
Pebax-2533/ZIF-7 50 wt%	6	1	8

**Table 4:** Gas permeation properties of two substrates.

Substrate	N_2_ Permeance (GPU)	N_2_ Error	CO_2_ Permeance (GPU)	CO_2_ Error	CO_2_/N_2_ Ideal Selectivity
PSf 18 wt%	800.87	2.18	1400.88	1.74	1.74
PSf 18 wt% + PEG 20,000 1 wt%	8422.11	16.73	14,300.06	1.70	1.70

**Table 5:** Gas permeance properties of Pebax-2533/ZIF-7 composite membranes.

Membranes	Selective Layer (µm)	N_2_ Permeability (GPU)	N_2_ Error	CO_2_ Permeability (GPU)	CO_2_ Error	CO_2_/N_2_ Ideal Selectivity
Pure Pebax-2533	1.35	2.68	0.73	18.43	1.24	6.88
Pebax-2533/ZIF-7 5 wt%	1.48	1.09	0.06	18.34	0.26	16.88
Pebax-2533/ZIF-7 15 wt%	1.42	0.53	0.07	21.48	0.03	40.68
Pebax-2533/ZIF-7 25 wt%	1.43	0.43	0.04	21.53	0.09	50.43
Pebax-2533/ZIF-7 35 wt%	1.61	0.71	0.02	26.22	0.18	37.04
Pebax-2533/ZIF-7 50 wt%	1.86	1.57	0.04	21.07	0.13	13.40

**Table 6:** Gas diffusivity properties of Pebax-2533/ZIF-7 composite membranes.

Membranes	N_2_ Diffusivity (10^−7^ cm^2^/s)	N_2_ Error	CO_2_ Diffusivity (10^−7^ cm^2^/s)	CO_2_ Error	CO_2_/N_2_ Diffusivity Selectivity
Pure Pebax-2533	96.41	24.50	196.03	10.60	2.03
Pebax-2533/ZIF-7 5 wt%	53.61	3.24	120.64	12.17	2.25
Pebax-2533/ZIF-7 15 wt%	52.78	13.89	118.00	8.22	2.24
Pebax-2533/ZIF-7 25 wt%	64.68	5.01	122.65	6.90	1.90
Pebax-2533/ZIF-7 35 wt%	46.29	4.92	88.55	6.95	1.91
Pebax-2533/ZIF-7 50 wt%	70.53	7.94	102.37	9.22	1.45

**Table 7:** Gas solubility properties of Pebax-2533/ZIF-7 composite membranes.

Membranes	N_2_ Solubility (10^−3^ cm^3^/cm^3^ atm)	CO_2_ Solubility (10^−3^ cm^3^/cm^3^ atm)	CO_2_/N_2_ Solubility Selectivity
Pure Pebax-2533	3.08	10.44	3.38
Pebax-2533/ZIF-7 5 wt%	2.19	16.45	7.50
Pebax-2533/ZIF-7 15 wt%	1.06	19.26	18.19
Pebax-2533/ZIF-7 25 wt%	0.83	22.01	26.60
Pebax-2533/ZIF-7 35 wt%	1.63	31.51	19.36
Pebax-2533/ZIF-7 50 wt%	2.52	23.25	9.23

**Table 8:** Gas permeation properties in various membranes with this work.

Membranes	CO_2_ Permeability (Barrer)	CO_2_/N_2_ Ideal Selectivity	Reference
Pure Pebax-2533	24.83	6.88	This work
Pebax-2533/ZIF-7 25 wt%	30.42	50.43	
Pebax-2533/Multiple metal-ion ZIFs 10 wt%	321	42.8	X. Zhang et al. [56]
Pebax-2533/ZIF-7-OH 8 wt%	249	32.5	J. Gao et al. [26]
nZIF-7 5 wt%/PEI	64.7	16.8	B.A. Al-Maythalony et al. [33]
Pebax-1657/PEI	11.09	28.44	M. Shafiee et al. [57]
Pebax-2533/PGO 0.02 wt%	397.35	23.75	R. Casadei et al. [48]

## Data Availability

Data presented in this study is contained within the article.

## References

[1] Masson-Delmotte V., Zhai P., Pörtner H.-O., Roberts D., Skea J., Shukla R., Pirani A., Okia M., Péan C., Pidcock R. (2018). Global Warming of 1.5 °C.

[2] Shin J.E., Han H.S., Ha S.Y., Park H.B. (2018). The State of the Art of Membrane Technologies for Carbon Dioxide Separation.

[3] Ahmadi M., Janakiram S., Dai Z., Ansaloni L., Deng L. (2018). Performance of mixed matrix membranes containing porous two-dimensional (2D) and three-dimensional (3D) fillers for CO_2_ separation: A review. Membranes.

[4] Robeson L.M. (1991). Correlation of separation factor versus permeability for polymeric membranes. J. Membr. Sci..

[5] Park H.B., Kamcev J., Robeson L.M., Elimelech M., Freeman B.D. (2017). Maximizing the right stuff: The trade-off between membrane permeability and selectivity. Science.

[6] Vinoba M., Bhagiyalakshmi Y., Alqaheem Y., Alomair A.A., Pérez A., Rana M.S. (2017). Recent progress of fillers in mixed matrix membranes for CO_2_ separation: A review. Sep. Purif. Technol..

[7] Dong G., Li H., Chen V. (2013). Challenges and opportunities for mixed-matrix membranes for gas separation. J. Mater. Chem. A.

[8] Liu M., Nothling M.D., Webley P.A., Fu Q., Qiao G.G. (2019). Postcombustion carbon capture using thin-film composite membranes. Acc. Chem. Res..

[9] Kattula M., Ponnuru K., Zhu L., Jia W., Lin H., Furlani E.P. (2015). Designing ultrathin film composite membranes: The impact of a gutter layer. Sci. Rep..

[10] Dai Z., Ansaloni L., Deng L. (2016). Recent advances in multi-layer composite polymeric membranes for CO_2_ separation: A review. Green Energy Environ..

[11] Ma Y., Shi F., Ma J., Wu M., Zhang J., Gao C. (2011). Effect of PEG additive on the morphology and performance of polysulfone ultrafiltration membranes. Desalination.

[12] Chakrabarty B., Ghoshal A., Purkait M. (2008). SEM analysis and gas permeability test to characterize polysulfone membrane prepared with polyethylene glycol as additive. J. Colloid Interface Sci..

[13] Fauzan N.A.B., Mannan H.A., Nasir R., Mohshim D.F.B., Mukhtar H. (2019). Various Techniques for Preparation of Thin-Film Composite Mixed-Matrix Membranes for CO_2_ Separation. Chem. Eng. Technol..

[14] Ma C., Wang M., Wang Z., Gao M., Wang J. (2020). Recent progress on thin film composite membranes for CO_2_ separation. J. CO2 Util..

[15] Shahrak M.N., Shahrak M.N., Shahsavand A., Khazeni N., Wu X., Deng S. (2017). Synthesis, gas adsorption and reliable pore size estimation of zeolitic imidazolate framework-7 using CO_2_ and water adsorption. Chin. J. Chem. Eng..

[16] He M., Yao J., Li L., Wang K., Chen F., Wang H. (2013). Synthesis of zeolitic imidazolate framework-7 in a water/ethanol mixture and its ethanol-induced reversible phase transition. Chem. Plus. Chem..

[17] Guan W., Dai Y., Dong C., Yang X., Xi Y. (2020). Zeolite imidazolate framework (ZIF)-based mixed matrix membranes for CO_2_ separation: A review. J. Appl. Polym. Sci..

[18] Noguera-Díaz A., Villarroel-Rocha J., Ting V.P., Bimbo N., Sapagb K., Maysa T.J. (2019). Flexible ZIFs: Probing guest-induced flexibility with CO_2_, N_2_ and Ar adsorption. J. Chem. Technol. Biotechnol..

[19] Phan A., Doonan J., Uribe-Romo F.J., Knobler C.B., O’keeffe M., Yaghi O.M. (2009). Synthesis, structure, and carbon dioxide capture properties of zeolitic imidazolate frameworks. Acc. Chem. Res..

[20] Zhao P., Lampronti G.I., Lloyd G.O., Wharmby M.T., Facq S., Cheetham A.K., Redfern S.A. (2014). Phase transitions in zeolitic imidazolate framework 7: The importance of framework flexibility and guest-induced instability. Chem. Mater..

[21] Zhao P., Lampronti G.I., Lloyd G.O., Suard E., Redfern S.A. (2014). Direct visualisation of carbon dioxide adsorption in gate-opening zeolitic imidazolate framework ZIF-7. J. Mater. Chem. A.

[22] Arami-Niya A., Birkett G., Zhu Z., Rufford T.E. (2017). Gate opening effect of zeolitic imidazolate framework ZIF-7 for adsorption of CH_4_ and CO_2_ from N_2_. J. Mater. Chem. A.

[23] Azizi N., Hojjati M.R. (2018). Using Pebax-1074/ZIF-7 mixed matrix membranes for separation of CO_2_ from CH_4_. Pet. Sci. Technol..

[24] Li T., Pan Y., Peinemann K., Lai Z. (2013). Carbon dioxide selective mixed matrix composite membrane containing ZIF-7 nano-fillers. J. Membr. Sci..

[25] Chakrabarty T., Neelakanda P., Peinemann K. (2018). CO_2_ Selective, Zeolitic Imidazolate Framework-7 Based Polymer Composite Mixed-Matrix Membranes. J. Mater. Sci. Res..

[26] Gao J., Mao H., Jin H., Chen C., Feldhoff A., Li Y. (2020). Functionalized ZIF-7/Pebax® 2533 mixed matrix membranes for CO_2_/N_2_ separation. Microporous Mesoporous Mater..

[27] Nafisi V., Hägg M. (2014). Development of dual layer of ZIF-8/PEBAX-2533 mixed matrix membrane for CO_2_ capture. J. Membr. Sci..

[28] Murali R.S., Ismail A.F., Rahman M.A., Sridhar S. (2014). Mixed matrix membranes of Pebax-1657 loaded with 4A zeolite for gaseous separations. Sep. Purif. Technol..

[29] Zheng W., Ding R., Yang K., Dai Y., Yan X., He G. (2019). ZIF-8 nanoparticles with tunable size for enhanced CO_2_ capture of Pebax based MMMs. Sep. Purif. Technol..

[30] Khoshkharam A., Azizi N., Behbahani R.M., Ghayyem M.A. (2017). Separation of CO_2_ from CH_4_ using a synthesized Pebax-1657/ZIF-7 mixed matrix membrane. Pet. Sci. Technol..

[31] Kim J., Park T., Chung E. (2021). Effect of 2-MeIM/Zn Molar Ratio on CO_2_ Permeability of Pebax/ZIF-8 Mixed Matrix Membranes. J. Membr. Sci. Res..

[32] Woo S.H., Park J., Min B.R. (2015). Relationship between permeate flux and surface roughness of membranes with similar water contact angle values. Sep. Purif. Technol..

[33] Al-Maythalony B.A., Alloush A.M., Faizan M., Dafallah H., Elgzoly M.A., Seliman A.A., Al-Ahmed A., Yamani Z.H., Habib M.A., Cordova K.E. (2017). Tuning the interplay between selectivity and permeability of ZIF-7 mixed matrix membranes. ACS Appl. Mater. Interfaces.

[34] Yeom C.K., Lee J.M., Hong Y.T., Kim S.C. (1999). Evaluation of Gas Transport Parameters through Dense Polymeric Membranes by Continuous-Flow Technique. Membr. J..

[35] Getie S., Belay A., Chandra Reddy A.R., Belay Z. (2017). Synthesis and characterizations of zinc oxide nanoparticles for antibacterial applications. J. Nanomed. Nanotechnol..

[36] Ebrahimi M., Mansournia M. (2017). Rapid room temperature synthesis of zeolitic imidazolate framework-7 (ZIF-7) microcrystals. Mater. Lett..

[37] Ebrahimi A., Mansournia M. (2018). Zeolitic imidazolate framework-7: Novel ammonia atmosphere-assisted synthesis, thermal and chemical durability, phase reversibility and potential as highly efficient nanophotocatalyst. Chem. Phys..

[38] Park K.S., Ni Z., Côté A.P., Choi J.Y., Huang R., Uribe-Romo F.J., Chae H.K., O’Keeffe M., Yaghi O.M. (2006). Exceptional chemical and thermal stability of zeolitic imidazolate frameworks. Proc. Natl. Acad. Sci. USA.

[39] Wu X., Shahrak M.N., Yuan B., Deng S. (2014). Synthesis and characterization of zeolitic imidazolate framework ZIF-7 for CO_2_ and CH_4_ separation. Microporous Mesoporous Mater..

[40] Cuadrado-Collados C., Fernández-Català J., Fauth F., Cheng Y.Q., Daemen L.L., Ramirez-Cuesta A.J., Silvestre-Albero J. (2017). Understanding the breathing phenomena in nano-ZIF-7 upon gas adsorption. J. Mater. Chem. A.

[41] Morris W., He N., Ray K.G., Klonowski P., Furukawa H., Daniels I.N., Houndonougbo Y.A., Asta M., Yaghi O.M., Laird B.B. (2012). A combined experimental-computational study on the effect of topology on carbon dioxide adsorption in zeolitic imidazolate frameworks. J. Phys. Chem. C.

[42] Lowell S., Shields J.E. (1991). Langmuir and BET theories (kinetic isotherms). Anonymous Powder Surface Area and Porosity.

[43] Guan P., Luo J., Li W., Si Z. (2017). Enhancement of gas permeability for CH_4_/N_2_ separation membranes by blending SBS to Pebax polymers. Macromol. Res..

[44] Azizi N., Hojjati M.R., Zarei M.M. (2018). Study of CO_2_ and CH_4_ permeation properties through prepared and characterized blended Pebax-2533/PEG-200 membranes. Silicon.

[45] Knozowska K., Li G., Kujawski W., Kujawa J. (2020). Novel heterogeneous membranes for enhanced separation in organic-organic pervaporation. J. Membr. Sci..

[46] Cai W., Lee T., Lee M., Cho W., Han D.Y., Choi N., Choi J. (2014). Thermal structural transitions and carbon dioxide adsorption properties of zeolitic imidazolate framework-7 (ZIF-7). J. Am. Chem. Soc..

[47] Dai Z., Bai L., Hval K.N., Zhang X., Zhang S., Deng L. (2016). Pebax^®^/TSIL blend thin film composite membranes for CO_2_ separation. Sci. China Chem..

[48] Casadei R., Giacinti Baschetti M., Yoo M.J., Park H.B., Giorgini L. (2020). Pebax^®^ 2533/Graphene Oxide Nanocomposite Membranes for Carbon Capture. Membranes.

[49] Wang Y., Alhassan S.M., Yang V.H., Schiraldi D.A. (2013). Polyether-block-amide copolymer/clay films prepared via a freeze-drying method. Compos. B Eng..

[50] O’Connor H.J., Dowling D.P. (2020). Comparison between the properties of polyamide 12 and glass bead filled polyamide 12 using the multi jet fusion printing process. Addit. Manuf..

[51] Xie K., Fu Q., Qiao G.G., Webley P.A. (2019). Recent progress on fabrication methods of polymeric thin film gas separation membranes for CO_2_ capture. J. Membr. Sci..

[52] Qian J., Wu T., Shi J., Chang H., Liu D., Pan Y. (2021). Improved CO_2_/CH_4_ separation performance of mixed-matrix membrane by adding ZIF-7-NH_2_ nanocrystals. J. Appl. Polym. Sci..

[53] Zhang L., Hu Z., Jiang J. (2012). Metal–organic framework/polymer mixed-matrix membranes for H_2_/CO_2_ separation: A fully atomistic simulation study. J. Phys. Chem. C.

[54] Pazirofteh M., Dehghani M., Niazi S., Mohammadi A.H., Asghari M. (2017). Molecular dynamics simulation and Monte Carlo study of transport and structural properties of PEBA 1657 and 2533 membranes modified by functionalized POSS-PEG material. J. Mol. Liq..

[55] Caro J. (2021). Diffusion coefficients in nanoporous solids derived from membrane permeation measurements. Adsorption.

[56] Zhang X., Zhang T., Wang Y., Li J., Liu C., Li N., Liao J. (2018). Mixed-matrix membranes based on Zn/Ni-ZIF-8-PEBA for high performance CO2 separation. J. Membr. Sci..

[57] Shafiee M., Akbari A., Foroutan R., Ramavandi B. (2020). The permeability and selectivity of nanocomposite membrane of PEBAx 1657/PEI/SiO_2_ for separation of CO_2_, N_2_, O_2_, CH_4_ gases: A data set. Data Brief.

